# Mechanisms of hippocampal sequence replay

**DOI:** 10.1186/1471-2202-16-S1-P11

**Published:** 2015-12-18

**Authors:** Paola Malerba, Giri P Krishnan, Maxim Bazhenov

**Affiliations:** 1 Cell Biology and Neuroscience, University of California Riverside, Riverside, CA 92507, USA

## 

Sleep is known to be important for memory consolidation [1], and memories are thought to be stored in the hippocampus during wakefulness and "transferred" to cortex during sleep [2]. Recently, memory replay - repeatable sequences of pyramidal cell firing - has been demonstrated during sleep, and associated with characteristic brain oscillations, giving rise to the hypothesis that these may form the critical neural substrate of memory consolidation. It is known that the content of hippocampal replay can be biased during sleep, in what is called cued-reactivation [3], and that similar paradigms show enhanced memory performance in humans [4]. Moreover, tampering with replay can disrupt memory formation and consolidation [5]. Despite extensive evidence highlighting the importance of replay within the broader phenomenon of sleep-mediated memory consolidation, the mechanisms underlying sequence replay are still unknown.

During sleep, replay events are associated with specific patterns of neuronal oscillations [6]. Replay is seen in cortex during slow oscillation - a rhythmic (< 1Hz) state in which periods of activity (active or Up states) alternate with quiet periods (silent or Down states), while replay in the hippocampus is associated with sharp-wave ripple events - irregularly brief bouts of high frequency (>150 Hz) firing, driven by strong excitatory inputs coming from CA3, which result in a strong deflection in the LFP of stratum radiatum (the sharp wave) [7].

In the present study, we build on our previous research [8] to develop a model of spike sequence replay during sleep. In the past, we have introduced a model of CA1 ripples in which oscillations are transients, mediated by the intrinsic frequency of CA1 basket cells driven by CA3 activation. In this work, we construct a model of CA3 area in which stochastic intrinsic activation of pyramidal cells originates a massive cell activation that results in a strong excitatory input to area CA1. We observe that sequential activation of a selected sub-group of CA1 pyramidal cells is driven by a less specific sequential activation in CA3. We then characterize the mechanisms underlying sequence selection and reactivation within a sharp-wave ripple event.

Using hippocampal and thalamocortical models, we investigate the role of cortical inputs on ripple timing and their specific spike content. Our study illustrates the possible role of cortical Up states during slow oscillation in biasing hippocampal replay, which is a core component of the cortico-hippocampal interaction underlying memory consolidation.
